# The Influence of Respiration on Blood Flow in the Fontan Circulation: Insights for Imaging-Based Clinical Evaluation of the Total Cavopulmonary Connection

**DOI:** 10.3389/fcvm.2021.683849

**Published:** 2021-08-05

**Authors:** Séline F. S. van der Woude, Friso M. Rijnberg, Mark G. Hazekamp, Monique R. M. Jongbloed, Sasa Kenjeres, Hildo J. Lamb, Jos J. M. Westenberg, Arno A. W. Roest, Jolanda J. Wentzel

**Affiliations:** ^1^Department of Cardiology, Biomedical Engineering, Biomechanics Laboratory, Rotterdam, Netherlands; ^2^Department of Cardiothoracic Surgery, Leiden University Medical Center, Leiden, Netherlands; ^3^Department of Anatomy, Embryology and Cardiology, Leiden University Medical Center, Leiden, Netherlands; ^4^Department of Chemical Engineering, Faculty of Applied Sciences, Delft University of Technology and J. M. Burgerscentrum Research School for Fluid Mechanics, Delft, Netherlands; ^5^Department of Radiology, Leiden University Medical Center, Leiden, Netherlands; ^6^Department of Pediatric Cardiology, Leiden University Medical Center, Leiden, Netherlands

**Keywords:** Fontan, total cavopulmonary connection, respiration - physiology, flow imaging, MRI, hepatic veins, blood flow, extracardiac conduit Fontan

## Abstract

Congenital heart disease is the most common birth defect and functionally univentricular heart defects represent the most severe end of this spectrum. The Fontan circulation provides an unique solution for single ventricle patients, by connecting both caval veins directly to the pulmonary arteries. As a result, the pulmonary circulation in Fontan palliated patients is characterized by a passive, low-energy circulation that depends on increased systemic venous pressure to drive blood toward the lungs. The absence of a subpulmonary ventricle led to the widely believed concept that respiration, by sucking blood to the pulmonary circulation during inspiration, is of great importance as a driving force for antegrade blood flow in Fontan patients. However, recent studies show that respiration influences pulsatility, but has a limited effect on net forward flow in the Fontan circulation. Importantly, since MRI examination is recommended every 2 years in Fontan patients, clinicians should be aware that most conventional MRI flow sequences do not capture the pulsatility of the blood flow as a result of the respiration. In this review, the unique flow dynamics influenced by the cardiac and respiratory cycle at multiple locations within the Fontan circulation is discussed. The impact of (not) incorporating respiration in different MRI flow sequences on the interpretation of clinical flow parameters will be covered. Finally, the influence of incorporating respiration in advanced computational fluid dynamic modeling will be outlined.

## Introduction

Congenital heart disease is the most common birth defect with an estimated incidence of 1 in 100 live births (1). Functionally univentricular heart defects represent the most severe end of the spectrum of congenital heart disease, characterized by a severely underdeveloped ventricle that is unable to drive the systemic or pulmonary circulation. Many underlying diagnoses can be present, including patients with an underdeveloped right ventricle (e.g., tricuspid atresia) or an underdeveloped left ventricle (e.g., hypoplastic left heart syndrome). The Fontan operation is the palliative treatment of choice for single ventricle patients, by connecting both caval veins directly to the pulmonary arteries (PAs), also called the total cavopulmonary connection (TCPC) (2). Via this procedure, the venous inflow connections to the heart are rerouted, excluding the hypoplastic ventricle from the circulation, whereas the other ventricle will serve as systemic ventricle. Without the interposition of a subpulmonary ventricle, the pulmonary circulation in Fontan patients is a low-energy, passive circulation that is dependent on elevated systemic venous pressure to drive pulmonary blood flow toward the single ventricle. The Fontan circulation is thus characterized by chronically elevated central venous pressure with reduced cardiac output due to chronic preload deprivation of the single ventricle (3). Although the Fontan circulation has led to survival into adulthood >90%, significant morbidity is present including a reduced quality of life, exercise capacity and the occurrence of liver fibrosis/cirrhosis or protein losing enteropathy (4).

Because of the vulnerable state of the Fontan physiology and its dependence on favorable hemodynamics, regular evaluation of flow within the Fontan circulation is recommended for early detection of (subclinical) complications (4). Currently, echocardiography and magnetic resonance imaging (MRI) are the imaging modalities of choice to evaluate TCPC flow, but differently incorporate the effect of respiration on flow rates. Since respiration importantly influences TCPC flow (5–7), knowledge about the effect of respiration on blood flow and how flow measurements and flow-related clinical parameters are affected by different MRI protocols is therefore important for clinicians taking care of Fontan patients.

In this review, blood flow characteristics as influenced by the cardiac and respiratory cycle at multiple locations within the TCPC are discussed. The effect of not/partially incorporating the influence of the respiratory cycle in conventional MRI flow imaging will be described. Finally, the importance of including respiration-resolved flow measurements in advanced computational fluid dynamic (CFD) modeling of TCPC hemodynamics (e.g., wall shear stress, energy loss) is outlined.

## The Total Cavopulmonary Connection: Definition of the Different Vessels

**Figure 1:**
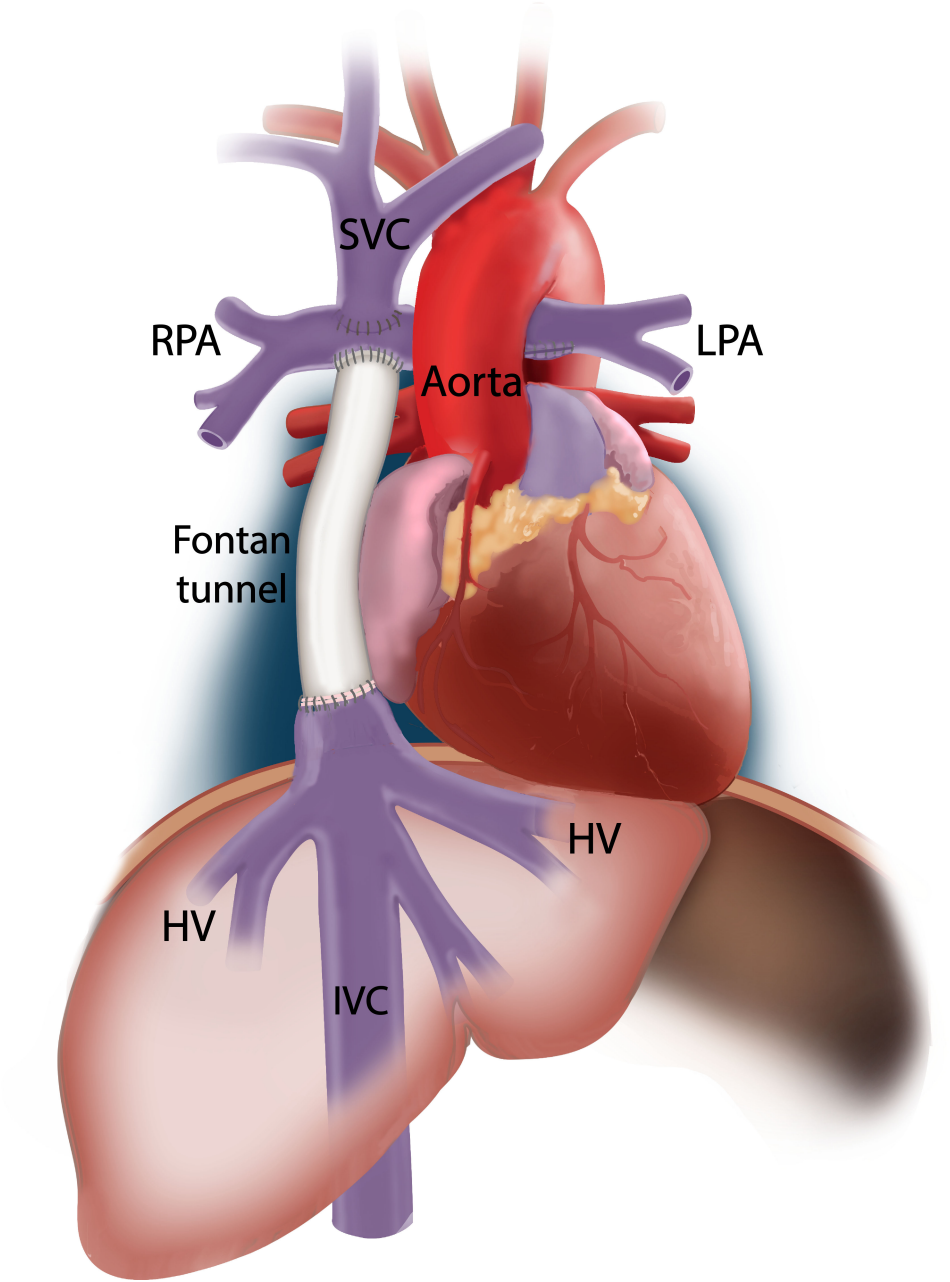
A schematic representation of the TCPC is shown. In this example, an extracardiac conduit Fontan circulation is shown. The IVC represents the part inferior to the entry of the hepatic veins. The suprahepatic part of the IVC is indicated as the Fontan tunnel, representing either the extracardiac conduit or lateral intraatrial tunnel.

Nowadays, the Fontan circulation is created using two techniques. The lateral tunnel technique connects the IVC to the PA via an intra-atrial patch (thus including part of the right atrium in the Fontan tunnel) (2). The extracardiac conduit technique (8) connects the IVC with the PA via a rigid Goretex conduit outside the heart. Conventionally, the term “TCPC” is considered to cover the area consisting of the Fontan tunnel (both the lateral tunnel and extracardiac conduit technique, above the entry of the HVs), the SVC, and both right- and left PAs. Thus, most papers use the terminology “IVC” when assessing flow in the Fontan tunnel (9–11). In this review, as depicted in Figure 1, the term “IVC” is used for the subhepatic IVC (below the entry of the HVs), and “Fontan tunnel” for the suprahepatic IVC (above the entry of the HVs) to make a clear distinction between these locations within the TCPC.

## Physiology of TCPC Flow

In the normal biventricular circulation, systemic venous return toward the right atrium is determined by the ratio of the pressure gradient between (1) the mean systemic filling pressure and right atrial pressure and (2) the venous vascular resistance (12). Consequently, a change in one of these parameters is needed to affect venous return and, as a consequence of the Frank-Starling mechanism, thereby affects preload leading to altered cardiac output. For example, factors that increase systemic filling pressure (e.g., augmented blood volume or vasomotor tone) can alter systemic venous return by increasing the pressure gradient promoting venous return (12, 13).

In the Fontan circulation, systemic venous return and thus pulmonary blood flow to the single ventricle is therefore determined by the ratio of (1) the pressure gradient between the mean systemic filling pressure and the atrium, and (2) the venous vascular resistance and the total resistance in the Fontan circuit, constituting of the serial TCPC resistance and pulmonary vascular resistance (3, 14). In general, four components have been described in literature that can affect flow rates in the TCPC, including alterations in blood flow along the cardiac cycle (15), the respiratory cycle, flow alterations because of peripheral muscular pump activity during lower-leg exercise (14, 16), and by gravitational forces, leading to decreased inferior systemic venous flow rates at the upright vs. the supine position (17). All these factors influence TCPC flow by altering the venous pressure gradient from the systemic veins toward the atrium. For example, central venous pressure has been shown to be raised to 20–30 mmHg during lower-leg exercise by the contribution of the peripheral muscle pump, thereby effectively raising the pressure gradient and thus pulmonary blood flow in Fontan patients (3, 18).

Unique to the Fontan circulation where the systemic venous return and pulmonary circulation are fully bypassed from the single ventricle, only minor alterations in blood flow occur along the cardiac cycle, with in general increased flow during systole and early diastole (early filling), with decreased flow during late diastole (atrial contraction) (15). In Fontan patients, however, the effect of respiration on venous return and pulmonary blood flow pulsatility is much more pronounced. This is predominantly caused by a change in intrathoracic pressures (i.e., intrapleural and pericardial pressures), leading to a change in atrial pressures, and a change in abdominal pressure, thereby effectively changing the pressure gradient from the inferior vena cava and hepatic veins, via the pulmonary arteries and pulmonary vascular bed, to the atrium. For this reason, mechanical ventilation using positive end-expiratory pressure has been shown to reduce cardiac output in Fontan patients (19), with significantly increased pulmonary blood flow and cardiac output when negative pressure ventilation is applied (19, 20). Additionally, it also explains reduced caval flow rates during the Valsalva maneuver, as increased intrathoracic pressures during Valsalva lead to a decreased pressure gradient and thus venous return (21).

Of note, it is currently not known if changes in PVR during respiration also have effect on the change in blood flow during respiration. In healthy persons, PVR is lowest around functional residual capacity, with an increase in PVR at total lung capacity or residual volume (22). To date, no studies have studied possible changes in PVR during normal respiration in Fontan patients.

The minor effect of the cardiac cycle on blood flow pulsatility is in contrast to a normal biventricular circulation, where a rise in pulmonary artery pressure during systole results in significant pulmonary blood flow alterations during the cardiac cycle, with a much less pronounced influence of respiration. Because of its relevance to blood flow imaging, flow dynamics along the cardiac and respiratory cycle are the subject of this review.

## Imaging Modalities for Assessment of TCPC Blood Flow Along Cardiac and Respiratory Cycle

### Doppler Echocardiography

Doppler echocardiography allows for real-time measurement of one-directional blood flow velocity along the direction of the ultrasound beam. Simultaneous recording of the respiratory and electrocardiography (ECG) signal provides insight into the timing of the velocity measurements with respect to the cardiac and respiratory cycle.

### Phase Contrast-MRI

Flow quantification using phase contrast MRI (PC-MRI) is based on the fact that changes in the phase of the MR signal along a magnetic field gradient are proportional to the velocity of the blood flow (23). Subsequently, flow rates can be calculated by multiplying the mean velocity over a cross section with the vessel cross-sectional area. For most PC-MRI techniques, however, the time to acquire these phase (i.e., velocity) images exceeds the length of a single heartbeat. Therefore, to obtain dynamic flow information along an entire cardiac cycle, it requires data acquisition over multiple heartbeats, gated to the ECG signal (ECG-gating). Subsequently, the data from multiple heartbeats is synchronized and retrospectively reconstructed into one single cardiac cycle. Importantly, flow imaging using ECG-gating will therefore not take respiratory effects on flow rates into account, as data from multiple different heartbeats are acquired irrespective of the phase of the respiratory cycle.

### Respiratory Motion Compensation

PC-MRI can be performed under breath hold (2D flow MRI only) or free-breathing conditions. Since breathing motion can lead to image artifacts with poor image quality, PC-MRI acquired during free-breathing usually requires some form of respiratory movement compensation. Most commonly, a respiratory abdominal belt or navigator is used to track the level of the diaphragm. Only data acquired within a predefined range around the end-expiratory diaphragm position is accepted to minimize breathing artifacts. Thus, only flow data acquired around the end-expiratory phase of the respiratory cycle will be captured when a respiratory navigator is used, similar to the breath-hold condition (24). Knowledge about the effect of respiration on blood flow and how flow measurements and flow-related clinical parameters are affected by the different PC-MRI sequences and respiratory compensation strategies is therefore important for clinicians taking care of Fontan patients.

Currently, multiple PC-MRI sequences are used that are mainly focused on flow dynamics during the cardiac cycle with variable degrees of incorporation of the respiratory component.

### 2D Flow MRI

2D flow MRI obtains ECG-gated, one-directional (through-plane) velocity at a predefined 2D plane at a vessel of interest, and is the current clinical standard for flow quantification (25). Scan durations are in the order of 10–15 s. 2D flow can be acquired using free-breathing, with or without respiratory motion compensation, or under breath-hold conditions. Thus, 2D flow MRI does not capture respiration induced flow variations.

### 2D Real-Time Flow MRI

Advances in MRI acquisition strategies nowadays allow for 2D real-time (ungated) flow acquisitions without the need for respiratory motion compensation, allowing for assessment of dynamic flow variations (typical temporal resolution 15–20 measurements per second) along both the cardiac and respiratory cycle (26). The respiratory and electrocardiography (ECG) signals are simultaneously recorded, allowing to synchronize the timing of the flow rate measurements with the phase of the cardiac and respiratory cycle.

### 3D Flow MRI

Recently, 3D flow MRI has been introduced for assessment of flow and flow-related parameters in the TCPC in Fontan patients, exploiting the negligible TCPC blood flow pulsatility along the cardiac cycle (27). With 3D flow MRI, three-directional velocities within a 3D volume of interest are acquired for a single, cardiac-cycle averaged (no ECG-gating) phase. It allows for quantification of cardiac-cycle averaged flow rates and flow related clinical parameters (e.g., pulmonary flow distribution), within a 1.5 min scan. 3D flow MRI does not incorporate the effect of respiration on flow characteristics (27).

### 4D Flow MRI

4D flow MRI allows for the acquisition of ECG-gated (usually 20–30 phases along the cardiac cycle), three-directional velocities within a 3D volume of interest. Flow rates can be retrospectively quantified at any vessel of interest within the scanned volume. Furthermore, it allows for visualization of three-dimensional flow patterns within the TCPC and quantification of advanced hemodynamic parameters (e.g., viscous energy loss rate). Scan durations are in the order of 8–16 min, depending on the application, sequence and use of respiratory motion compensation (28). Due to the long scan times, 4D flow MRI can only be acquired using free-breathing with or without the use of respiratory motion compensation. Consequently, conventional 4D flow MRI sequences do not incorporate the effect of respiration on flow dynamics.

### 5D Flow MRI

Most recently, 5D flow MRI has been used to quantify blood flow by obtaining ECG- and respiratory-gated, three-directional velocities within a 3D volume (i.e., 4D flow MRI + respiratory-gating = 5D flow MRI). 5D flow MRI allows for obtaining cardiac-cycle resolved (ECG-gated) flow information from data acquired in four different respiration phases: inspiration, end-inspiration, expiration and end-expiration (29).

## The Influence of the Cardiac and Respiratory Cycle on Net Forward Flow in the TCPC

An important differentiation must be made between net forward flow and flow pulsatility within the TCPC. It is a common belief that part of the venous return in Fontan patients is dependent on energy provided by respiration (9, 17). Hsia et al. (17) used doppler echocardiography to define the percentage of respiration-dependent flow as follows: $\frac{Qinsp-Q\exp}{Qinsp+Qexp}$, where Q_insp_ and Q_exp_ are the flow rates during inspiration and expiration, respectively. Based on this parameter, ~30, 14, and 55% of Fontan tunnel flow, subhepatic IVC and HV flow is respiration-dependent, respectively (30). In comparison, in healthy controls respiration-dependent flow in the subhepatic IVC, HVs, and suprahepatic IVC were 11, 25, and 15%, respectively. Therefore, it has been suggested that a significant percentage of specifically Fontan tunnel and HV net forward flow is dependent on respiration as a driving force. However, although inspiration does actively *increase* flow rates compared to breath-holding conditions due to a decrease in intrathoracic pressure and atrial pressure augmenting the transpulmonary pressure gradient, it must be emphasized that expiration also leads to *decreased* flow rates, thereby mostly countering the effect of inspiration. Consequently, defining the respiration-dependency of flow using inspiration and expiration flow rates is not ideal. In fact, respiration has only a significant influence on the net forward flow if the increased flow volume during inspiration (i.e., inspiratory flow rate ^*^ duration of inspiration) outweighs the decreased flow volume during expiration, compared to a breath-hold condition. Indeed, Wei et al. (11) demonstrated that respiration did not significantly affect net forward flow rates (difference on average <0.1 L/min/m^2^) by comparing 2D real-time MRI acquired under both free-breathing and breath-hold conditions in the SVC, Fontan tunnel and aorta. Recently, Gabbert et al. also stressed the limited influence of respiration on net forward flow in the Fontan tunnel (7). In line with these findings, net forward flow was not affected by forced breathing conditions or after a 6 week inspiratory muscle training program (5, 31). Initiation of hyperventilation (representing the ventilatory pump) in addition to zero resistance exercise (representing the muscle pump) did also not result in significantly higher blood flow rates (16). Thus, it must be concluded that respiration is not an important driving force for net forward flow in the TCPC.

## The Influence of the Cardiac and Respiratory Cycle on Blood Flow Pulsatility in the TCPC

However, these studies clearly showed a significant increase in *pulsatility* (flow variations along the cardiac and/or respiratory cycle) caused by respiration, with high variability between the different vessels of the TCPC (5, 7, 11). Various parameters have been used to assess blood flow pulsatility along the respiratory cycle, of which the *inspiratory-to-expiratory flow ratio* (mean inspiratory flow rate divided by mean expiratory flow rate, Q_insp_/Q_exp_) is most commonly used.

**Figure 2:**
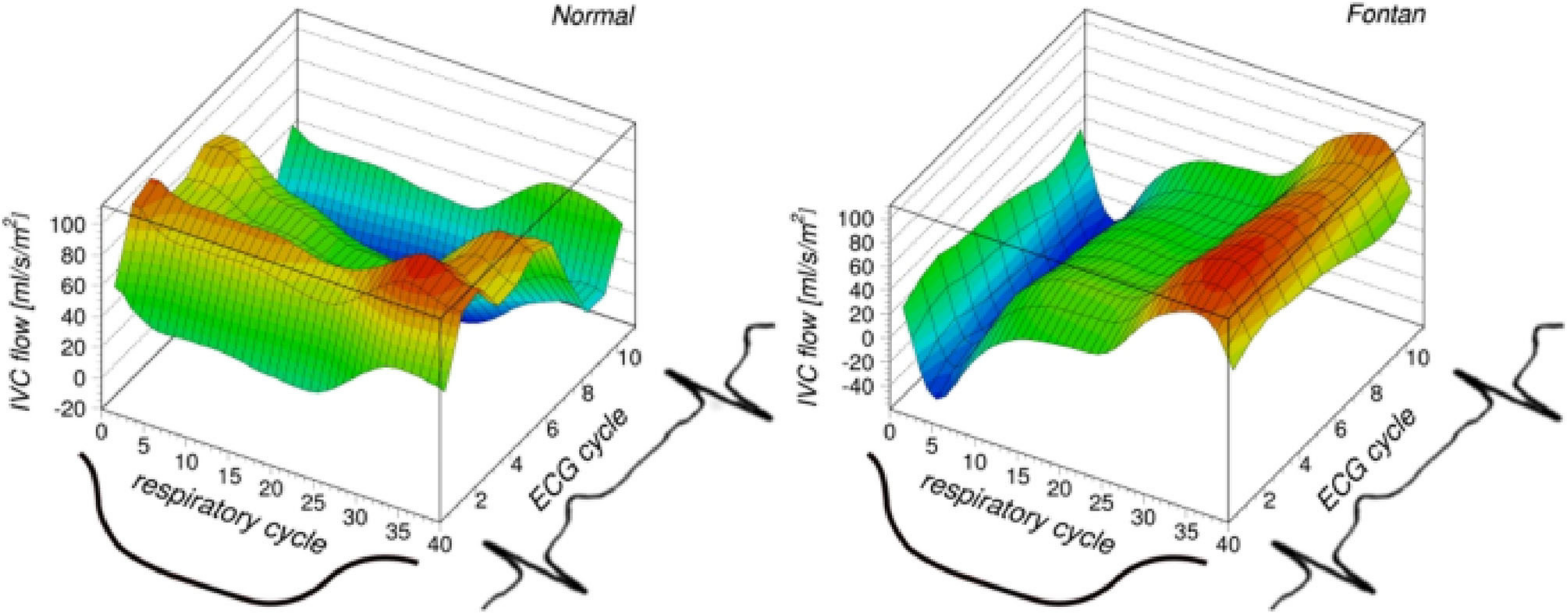
Reproduced from Gabbert et al. (7) https://www.nature.com/articles/s41598-019-38848-5/figures/2. This figure shows the blood flow profile in the suprahepatic IVC (healthy control, left image) and Fontan tunnel (right image), separated along the respiratory and cardiac cycle. Flow rates are indicated in color, with red indicating higher flow rates and blue indicating lower/negative flow rates. In healthy controls, pulsatility is almost exclusively observed along the cardiac cycle with retrograde flow observed during atrial contraction. In complete contrast, in Fontan patients pulsatility is almost exclusively observed along the respiratory cycle, with minimal or negative flow rates observed during expiration and maximal flow rates during inspiration. Along the cardiac cycle, negligible pulsatility is present.

Fontan tunnel flow represents ~65–70% of total systemic venous return (9, 32). Many studies consistently show the pronounced effect of respiration on Fontan tunnel flow rates (5, 9–11, 30, 33), with a 70–90% higher flow rate during inspiration compared to the entire respiratory cycle, significantly more pronounced compared to a 20% increase observed in healthy controls (5, 9). Other studies demonstrate similar findings by reporting a Q_insp_/Q_exp_ of 1.6–3.0 (11, 34, 35). By dividing 2D real-time flow MRI measurements of the Fontan tunnel into components along the respiratory and cardiac cycle, it was shown that respiration-derived pulsatility was 2.8-times the pulsatility along the cardiac cycle. This was the opposite in healthy controls, where pulsatility along the cardiac cycle was 2.5-times the pulsatility along the respiratory cycle at the level of the suprahepatic IVC (Figure 2) (7). As opposed to its effect on net forward flow, normal and forced breathing significantly increases blood flow pulsatility in Fontan patients, further illustrating that respiration rather than the cardiac cycle is the major contributor of flow pulsatility in the Fontan tunnel (Figure 2) (5, 7, 11).

The Fontan tunnel receives blood from both the subhepatic IVC and HVs, which contribute on average 62 and 38%, respectively, to total Fontan tunnel flow (6, 17, 35–38). The study of flow dynamics in this area is of great importance, as the splanchnic venous return plays an important role in the pathophysiology of liver cirrhosis and protein-losing enteropathy. Using (invasive) doppler echocardiography, Q_insp_/Q_exp_ in the subhepatic IVC was 1.3–1.6 (17, 35), not significantly different from healthy controls (Q_insp_/Q_exp_ 1.2) (6, 17, 36, 37). Thus, respiration has a much less pronounced influence at the subhepatic IVC compared to the Fontan tunnel, indicating that most of the respiratory-derived pulsatilty in the Fontan tunnel must be explained by the HV flow contribution.

Respiration indeed strongly influenced HV flow in Fontan patients (Q_insp_/Q_exp_ 2.9–4.4), significantly higher compared to healthy controls (Q_insp_/Q_exp_ 1.7) (17, 35). The important increase in HV flow during inspiration is explained by the liver acting as a reservoir of blood with high venous capacitance, from which both the increased extra- to intra-thoracic venous pressure gradient, as well as the direct pressure of the diaphragmatic descent on the liver, can draw blood toward the Fontan tunnel during inspiration (39). Presence of a fenestration was associated with a significantly higher inspiratory-to-expiratory fraction; 4.4 vs. 3.0 (36). Since central venous pressure is lowered by the presence of a fenestration between the Fontan tunnel and the atrium, the decreased afterload for HV flow likely causes the increased flow rates during inspiration. Importantly, plication of the diaphragm in Fontan patients with a diaphragm paresis does not fully restore normal respiratory mechanics, evidenced by a significantly smaller inspiratory-to-expiratory ratio of HV flow; 2.3 vs. 3.2 (37). However, respiration likely also does not affect net forward HV flow, in line with observations in the Fontan tunnel. This might explain why Fontan patients with a diaphragm paresis have similar cardiac index and exercise capacity compared to patients with a normal functioning diaphragm (40).

Studies on the influence of respiration on SVC flow, contributing ~35% of total systemic venous return, have been conflicting (Q_insp_/Q_exp_ 1.0–1.9) (5, 9, 11, 35). Wei et al. reported an inspiratory-to-expiratory ratio of 1.9 in the SVC, higher compared to the fraction of 1.6 they observed in the Fontan tunnel (11). This is in strong contrast to a previous study using 2D real-time flow MRI, which did not find an effect of breathing on SVC flow rates, in line with observations in healthy controls (5, 9). It remains the question from which vascular region with high venous capacitance (analog to the HVs providing most of the pulsatility observed in the Fontan tunnel) blood would be drawn toward the SVC during inspiration.

**Figure 3:**
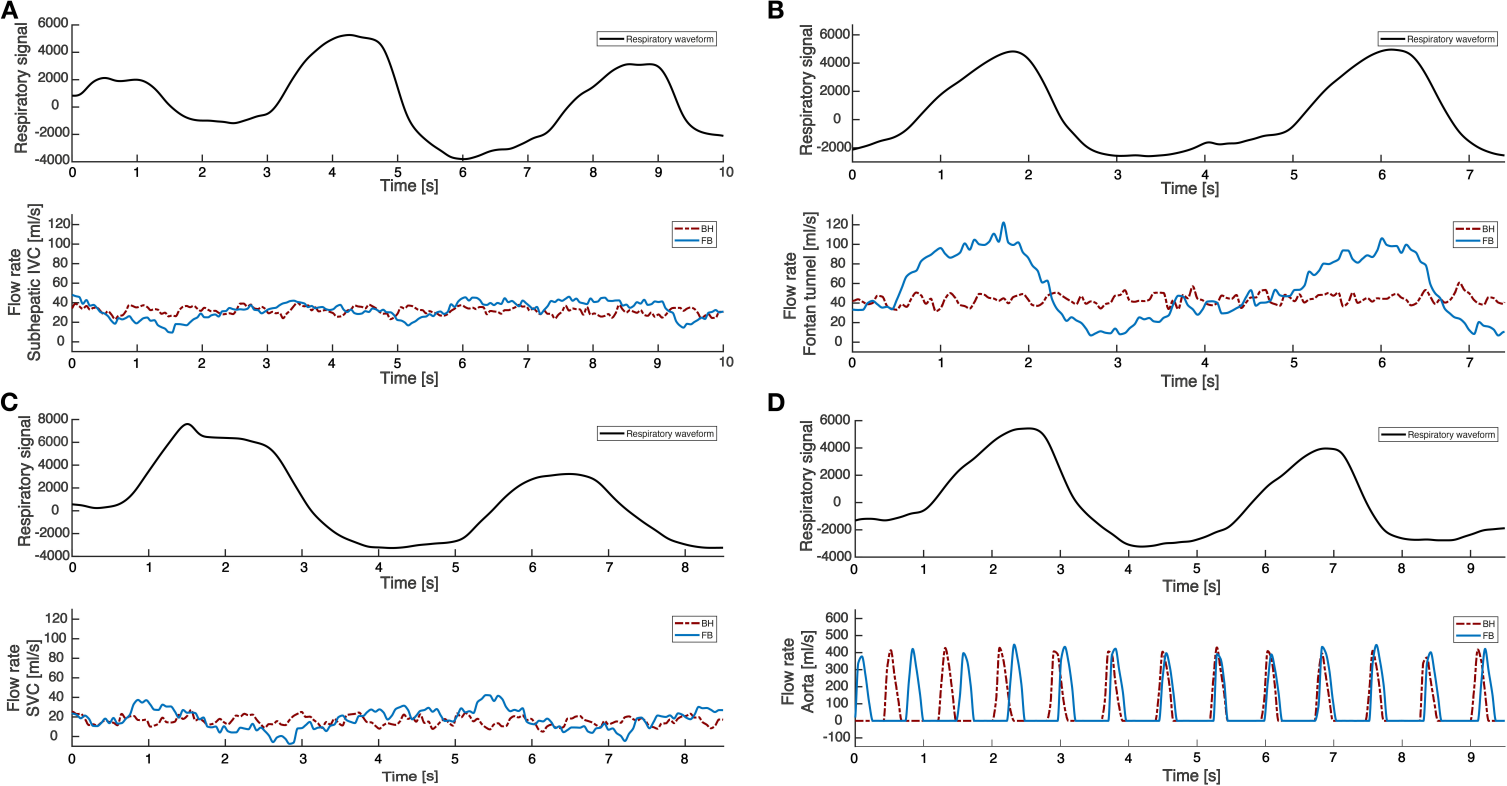
2D real-time flow MRI measurements in the IVC, Fontan tunnel, SVC and aorta. 2D real-time flow MRI measurements are shown under both free breathing (FB, blue line) and breath-hold (BH, red line) conditions for the subhepatic IVC **(A)**, Fontan tunnel **(B)**, SVC **(C)**, and ascending aorta **(D)**. Under breath-hold conditions, negligible cardiac-cycle related pulsatility is present in all venous vessels due to the absence of a subpulmonary ventricle in Fontan patients. During free breathing conditions, predominantly the Fontan tunnel flow is influenced by respiration, with a significant augmentation of flow rates during inspiration compared to expiration. Since the effect of respiration on subhepatic IVC flow is only minimal, most respiratory pulsatility in the Fontan tunnel originates from the hepatic veins. The influence of respiration on flow rates in the SVC and aorta is also minimal, although studies have reported conflicting results regarding respiratory pulsatility in the SVC.

**Figure 4:**
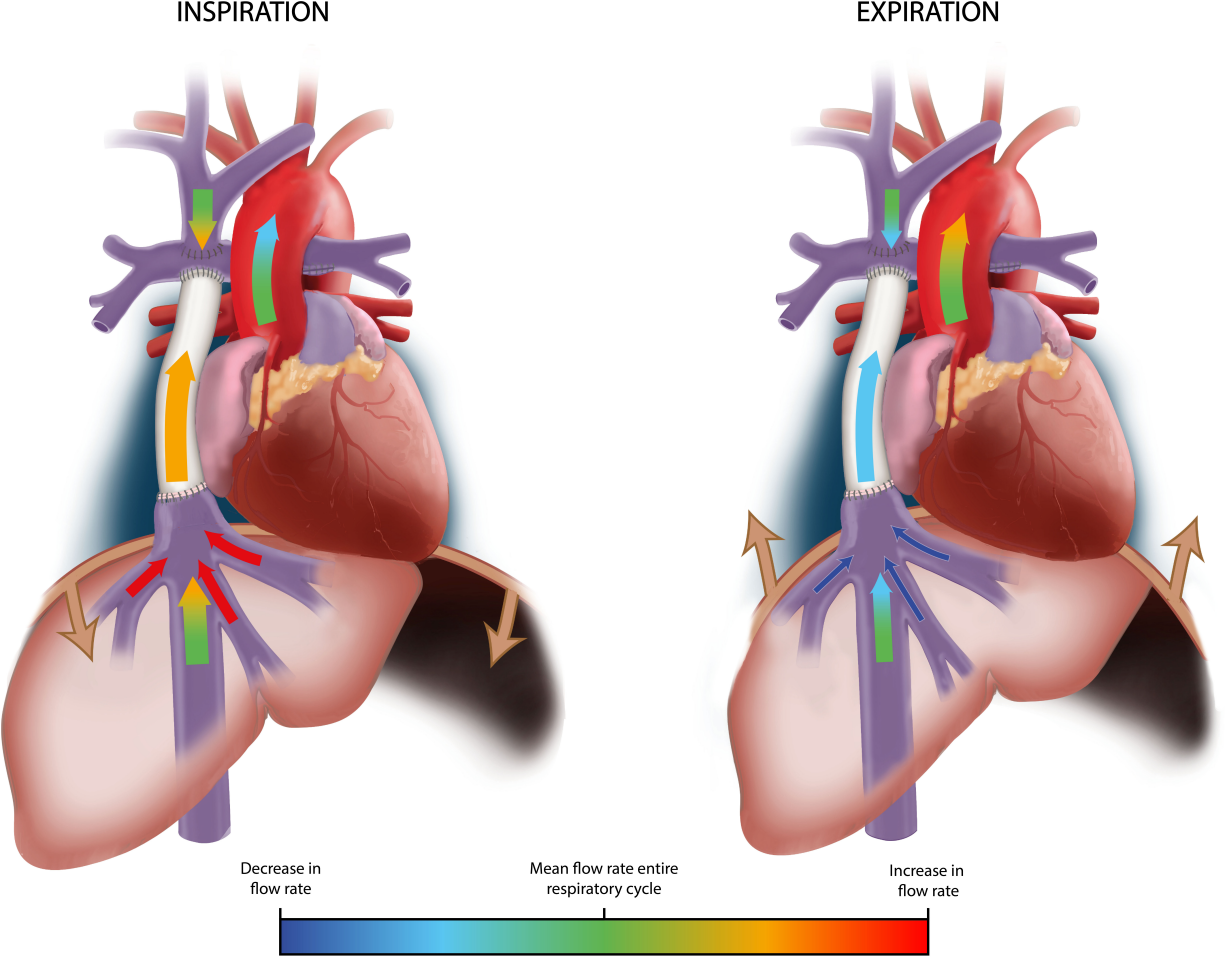
Schematic representation of the influence of respiration on flow rates in the TCPC and aorta. A schematic representation of flow rates in the TCPC and aorta is shown during inspiration (left image) and expiration (right image). Arrows are color-coded by the change in flow rate in each vessel during inspiration and expiration using a linear gradient toward red (strong increase) or dark blue (strong decrease), compared to the average flow rate during the entire respiratory cycle (light green). Change in width of the arrows for each vessel schematically represent the change in flow rates between inspiration and expiration. Flow rates in the subhepatic IVC, SVC* and aorta are only minimally influenced by respiration. Predominantly HV flow is strongly influenced by respiration, with a significant increase in flow rate during inspiration and decrease during expiration. Therefore, Fontan tunnel flow rates also show a considerable increase in flow rate during inspiration and decrease during expiration, primarily caused by the contribution from the HVs. *Of note, studies report conflicting results regarding respiratory pulsatility in the SVC, ranging from only minimal to considerable pulsatility. SVC/IVC, superior/inferior vena cava; HV, hepatic veins.

In normal subjects, aortic flow rate or ventricular stroke volume only slightly increase during expiration and decreases during inspiration, opposite to systemic venous return. Compared to mean aortic flow rates, flow rates are 0–6% higher at (end)-expiration and 1–6% lower at (end)-inspiration (5). A similar, modest effect of respiration on aortic flow has been observed in Fontan patients, ranging from a 7% increase during expiration, to a 4% decrease during inspiration (5, 9). Therefore, since systemic venous flow (predominantly HV and Fontan tunnel flow) rates are markedly raised during inspiration while aortic flow rates are not, the pulmonary circulation and lungs act as a reservoir with a large inspiratory capacity, releasing blood toward the single ventricle during expiration (9). In contrast to the systemic venous part of the Fontan circulation, respiration thus has a minimal influence on pulsatility in the aorta, which is primarily caused by the cardiac contraction. An example of flow rates during free breathing and under breath-hold conditions in the subhepatic IVC, HVs, Fontan tunnel and aorta are shown in Figure 3. An overview of the influence of respiration on flow rates and a schematic representation are presented in Table 1 and Figure 4.

**Table 1:** Influence of respiration on blood flow at multiple locations within the Fontan circulation.

**Parameter**	**Fontan**					**Healthy**				
	**Fontan tunnel**	**Subhepatic IVC**	**HV**	**SVC**	**Aorta**	**Suprahepatic IVC**	**Subhepatic IVC**	**HV**	**SVC**	**Aorta**
Inspiratory-to-expiratory flow ratio: Q_insp_/Q_exp_	1.6–3.0	1.3–1.6	2.9–4.4	1.0–1.9			1.2	1.7	1.2	
Inspiratory flow fraction: Q_insp_/Q_avg_	1.7–1.9	–	–	–	0.96	1.2	–	–	–	0.94–0.99
Respiratory-dependent flow fraction: (Q_insp_-Q_exp_)/(Q_insp_+Q_exp_)	30%	14%	55%	–	–	15%	11%	25%	–	–

*IVC/SVC, inferior/superior vena cava; HV, hepatic veins; Q_insp_, inspiratory flow rate; Q_exp_, expiratory flow rate; Q_avg_, flow rate during the entire respiratory cycle*.

### Clinical Relevance of Pulsatility

It is currently not known if respiratory derived pulsatility plays an important role in maintaining low pulmonary vascular resistance. The negligible blood flow pulsatility along the cardiac cycle has been thought to negatively influence pulmonary vascular resistance and endothelial function in Fontan patients, by altering the passive recruitment of capillaries and shear stress-mediated nitric oxide release (41). In turn, the respiratory derived pulsatility in the Fontan tunnel is profoundly different in amplitude and frequency compared to the cardiac pulsatility (Figure 3B). Recently, no significant difference was found in pulmonary vascular resistance between Fontan patients with or without diaphragm paresis, indicating that respiration derived pulsatility might not be important for healthy pulmonary vasculature (40).

### Retrograde Flow

While flow rates increase during inspiration, flow rates in the Fontan tunnel strongly decrease during expiration with potential back flow during the early expiratory phase. On average, backflow represents ~5–11% (range 0–30%) of mean forward flow volume in the Fontan tunnel (7, 9, 10), reducing to only 2.9% under exercise conditions (9). Similar percentages are observed in the suprahepatic IVC in healthy controls with a mean backflow of 6% (range 0–20%). Other studies used the retrograde-to-forward flow rate ratio to express back flow, but this parameter does not represent retrograde-to-antegrade flow *volume*, as the duration of antegrade and retrograde flow are not taken into account. In the subhepatic IVC and HVs, a retrograde-to-forward flow rate ratio of 0.06–0.07 and 0.27, respectively, were observed (17, 34, 36). Importantly, backflow is also observed in healthy persons as a result of the atrial contraction and is not related to respiration, while backflow in Fontan patients is related to the expiratory phase (7, 38). Retrograde flow is usually negligible in the SVC, accounting for only 0–1% of mean forward flow volume (9).

## The Influence of Respiration on Blood Flow During Exercise

Currently, the influence of respiration on flow rates under exercise conditions in Fontan patients has only been investigated in the Fontan tunnel, SVC and aorta. During lower-leg exercise conditions, the predominance of inspiratory flow augmentation in the Fontan tunnel seems to become less pronounced. Hjortdal et al. found a mean inspiratory fraction (Q_insp_/Q_average respiratory cycle_) decrease from 1.9 in rest, to 1.4 under 1 W/kg lower-leg exercise conditions. Peripheral muscle contractions were responsible for an almost 3-fold increase in expiratory flow rates, while inspiratory flow rates only increased by 1.6-fold (10). Of interest, Cordina et al. also showed predominantly increased expiratory flow rates in rest in patients after 20 weeks of peripheral muscle resistance training. The increased flow rates are explained by the presence of increased peripheral muscle mass leading to reduced venous compliance, thereby presumably increasing systemic filling pressures leading to increased preload and cardiac output (21). The reason why the muscle pump predominantly increases expiratory flow rates compared to inspiratory flow rates, thereby leading to reduced “respiratory dependence,” remains incompletely understood. Although speculative, there may be a limit in the venous capacity in the inspiratory phase, leading to a diminished response to increased filling pressures caused by the muscle pump.

## Effect of Respiration on Clinically Used Flow Parameters

### PC-MRI Derived Clinical Flow Parameters

Previous paragraphs have shown the variable influence of respiration on flow rates observed at multiple locations in the TCPC. Most conventional PC-MRI sequences are focused to capture flow changes during the cardiac contraction (ECG-gating) only and do not take respiration into account. Therefore, knowledge about the effect of (not) incorporating respiration on flow and flow-related clinical measurements are important for the correct interpretation of MRI examinations in Fontan patients.

### Net Forward Flow vs. Pulsatility

Since respiration predominantly affects pulsatility but not net forward flow (7, 11), conventional ECG-gated 2D flow MRI sequences did not show significantly different net forward flow rates between patients scanned under free-breathing vs. breath-hold conditions (42). However, dynamic flow characteristics specific to part of the respiratory cycle, such as retrograde flow during expiration, or peak velocities during inspiration will not be captured (7, 43). The same likely applies for ECG-gated sequences using a respiratory navigator (as the acceptance window contains the last part of expiration, the end-expiratory phase and the first part of inspiration), although no studies exist in Fontan patients comparing free-breathing navigator-gated PC-MRI with PC-MRI acquisitions during breath-hold conditions.

### Net Forward Flow-Derived Flow Parameters

Clinical parameters derived from net forward flow rates can thus most likely be accurately determined using ECG-gated sequences, since respiration minimally affects net forward flow rates. Only limited data exist of studies that have evaluated the effect of respiration on these parameters. Using a 4D flow MRI sequence able to provide cardiac-cycle resolved flow data based on inspiratory or expiratory data only, Rutkowski et al. showed a non-significant difference in pulmonary flow distribution (52 vs. 64% based on expiratory and inspiratory data, respectively) (44). Modeling studies using computational fluid dynamics (CFD) found similar results, with differences <5% in right-to-left pulmonary flow distribution along the respiratory cycle (45, 46).

### Hepatic Flow Distribution

Fontan patients require a certain amount of hepatic venous flow toward both lungs in order to prevent the formation of pulmonary arteriovenous malformations (47). The hepatic flow distribution (HFD) can be determined by tracking particles from the Fontan tunnel toward the PAs based on time-resolved, three-dimensional velocity fields acquired with 4D- (48, 49) or 5D flow MRI (29), or derived from computational fluid dynamic (CFD) models (39, 50, 51). Bastkowski et al. used a novel 5D flow MRI sequence to reconstruct four ECG-gated flow fields using data from 4 respiratory phases: inspiration, end-inspiration, expiration and end-expiration. On average, the maximum differences in HFD between the four respiratory-phases was 20% (range 9–30%). Hence, the contribution of hepatic flow toward the PA changes during the respiratory cycle because of the flow pulsatility in the Fontan tunnel associated with respiration. However, the necessity of including respiration for accurate average HFD quantification was not investigated (29). A recent study using patient-specific CFD models with both 2D real-time MRI acquired under free-breathing and breath-hold as boundary conditions, showed that respiration has negligible influence on average HFD with mean differences of 1% (range −3 to 7%) (43).

### CFD-Derived Hemodynamic Metrics of the TCPC

CFD models are increasingly used to study flow dynamics in the TCPC in Fontan patients, and can now be performed using patient-specific 3D TCPC reconstructions and patient-specific physiological data. It allows not only for the visualization of time-resolved 3D flow patterns within the TCPC, but also for quantification of advanced velocity and pressure-related hemodynamic parameters, including power loss, viscous energy loss rate, wall shear stress and stagnation volume (52–54).

### Importance of Including Respiration-Derived Pulsatility in CFD Simulations

#### Flow Patterns

Previous studies using CFD, *in vitro* models or 4D flow MRI have shown the presence of adverse secondary flow patterns, including helical, swirling flow patterns at the IVC-to-conduit junction (55) and in the PAs (56, 57) or caval flow collision leading to chaotic flow disturbances at the central Fontan confluence (58, 59). The appearance of adverse, energy-consuming flow patterns increase blood flow resistance that may lead to an increased risk of complications (14). Importantly, these dynamic, 3D flow patterns change when respiration is included (43, 60). Furthermore, potential deleterious effects of backflow on the splanchnic circulation and its association with liver fibrosis can only be studied by incorporating respiration into the models, as backflow is exclusively observed during (early) expiration. In addition, incorporation of respiration may also be important to study whether the pulsatile HV hemodynamics effect the presence and magnitude of local secondary flow patterns observed in the IVC-to-conduit junction using 4D flow MRI (55).

#### Energy Loss

Power loss and viscous energy loss describe the flow efficiency in the TCPC in Fontan patients which is related to the presence of adverse flow patterns and geometries (e.g., PA stenosis or undersized extracardiac conduit) (61, 62). Increased power loss and TCPC resistance have been associated with reduced exercise capacity (63) and increased levels of liver fibrosis (64). Inclusion of respiration is important for accurate power loss measurements, as incorporation of respiration resulted in a 1.4–3.1-fold increase in power loss, consistently higher compared to simulations incorporating the cardiac cycle only (43, 45, 60, 65).

Importantly, although not incorporating respiration in CFD models leads to an underestimation of power loss, it did not affect the ranking of multiple surgical TCPC options created using “virtual surgery” CFD platforms (66). Virtual surgery platforms allow for the pre-operative determination of the optimal TCPC geometry by evaluating the flow efficiency and HFD within the proposed TCPC using patient-specific CFD simulations (67).

#### Thrombosis Markers

Thrombosis can occur in some Fontan patients within the TCPC. Although no clear markers can currently predict thrombosis risk, regions with low wall shear stress and/or high stagnation volumes have been reported as potential markers. Stagnation volume (blood volume with a velocity <0.01 m/s) can be specifically high during expiration in large conduits with significantly reduced flow stagnation during inspiration (53). Thus, it is emphasized that incorporation of respiration is important when local hemodynamic metrics are of interest, including wall shear stress and stagnation volume, due to the high temporal variation of such metrics during the respiratory cycle (45, 53, 65). As a result, inclusion of pulsatile boundary conditions acquired under free-breathing in patient-specific CFD models is recommended (11, 60, 68).

## Conclusion

In conclusion, in contrast to the healthy circulation, respiration is the main source of blood flow pulsatility in the TCPC, whereas cardiac contraction mostly drives the net forward flow rate. Consequently, conventional ECG-gated PC-MRI acquisitions (i.e., 2D flow MRI and 4D flow MRI) can be used for measurements of clinical parameters based on net forward flow.

Inclusion of respiratory pulsatility in state-of-the-art patient-specific CFD models are recommended for evaluation of detailed, time-resolved hemodynamic metrics (e.g., wall shear stress and viscous energy loss rate), continuing to provide important insights for clinicians in the functioning of the TCPC.

## Author Contributions

SW performed the initial literature review. FR drafted the first manuscript. All authors contributed to manuscript revision, read, and approved the submitted version.

## Conflict of Interest

The authors declare that the research was conducted in the absence of any commercial or financial relationships that could be construed as a potential conflict of interest.

## Publisher's Note

All claims expressed in this article are solely those of the authors and do not necessarily represent those of their affiliated organizations, or those of the publisher, the editors and the reviewers. Any product that may be evaluated in this article, or claim that may be made by its manufacturer, is not guaranteed or endorsed by the publisher.
